# Biclustering fMRI time series: a comparative study

**DOI:** 10.1186/s12859-022-04733-8

**Published:** 2022-05-23

**Authors:** Eduardo N. Castanho, Helena Aidos, Sara C. Madeira

**Affiliations:** LASIGE, Faculdade de Ciências, Universidade de Lisboa, Lisbon, Portugal

**Keywords:** Biclustering, fMRI, Neurosciences, Time series analysis

## Abstract

**Background:**

The effectiveness of biclustering, simultaneous clustering of rows and columns in a data matrix, was shown in gene expression data analysis. Several researchers recognize its potentialities in other research areas. Nevertheless, the last two decades have witnessed the development of a significant number of biclustering algorithms targeting gene expression data analysis and a lack of consistent studies exploring the capacities of biclustering outside this traditional application domain.

**Results:**

This work evaluates the potential use of biclustering in fMRI time series data, targeting the Region × Time dimensions by comparing seven state-in-the-art biclustering and three traditional clustering algorithms on artificial and real data. It further proposes a methodology for biclustering evaluation beyond gene expression data analysis. The results discuss the use of different search strategies in both artificial and real fMRI time series showed the superiority of exhaustive biclustering approaches, obtaining the most homogeneous biclusters. However, their high computational costs are a challenge, and further work is needed for the efficient use of biclustering in fMRI data analysis.

**Conclusions:**

This work pinpoints avenues for the use of biclustering in spatio-temporal data analysis, in particular neurosciences applications. The proposed evaluation methodology showed evidence of the effectiveness of biclustering in finding local patterns in fMRI time series data. Further work is needed regarding scalability to promote the application in real scenarios.

## Background

fMRI data is used to evaluate connectivity in the brain, i.e., how brain regions interact together over time. When compared to other neuroimaging techniques, such as Electroencephalography (EEG) and Magnetoencephalography (MEG) (known to have a great temporal resolution), fMRI has a high spatial resolution while maintaining a temporal resolution of a few seconds [1, 2].

There are several strategies for fMRI data analysis, such as independent component analysis, machine learning, graph theory, among other [3]. These strategies require different modeling options for the data, such as 4-dimensional arrays (capturing the three region dimensions and the temporal one), slices of figures or multivariated time series [4, 5]. In our study, we consider the use of unsupervised machine learning techniques to analyse multivariated fMRI time series. The most popular form of unsupervised machine learning is clustering, which uses the similarity between features (columns) to partition a set of subjects (rows) into groups (clusters).

Traditional clustering approaches are rigid since they search for similarities considering all features of the subjects and do not allow a subject to belong to multiple groups [6]. Biclustering algorithms were proposed to overcome these limitations of clustering by clustering the rows and columns of a data matrix simultaneously [7]. Biclustering has been widely studied over the last 20 years following the development of the Chen and Church algorithm [8]. Multiple algorithms and computational frameworks for biclustering were developed [9], and biclustering is used in domains such as biomedicine, text mining, and marketing analysis [7, 10].

Despite these uses, the main application scenario of biclustering is the traditional gene expression context, which generates a bias in the development and application of biclustering. This bias has two main consequences: **first**, gene expression datasets act as a benchmark both during the development of new biclustering algorithms and in their independent comparison, meaning that these new algorithms are not compared in contexts other than this specific problem. **Second**, results in real datasets are usually compared using measures of biological relevance, such as the Gene Ontology (GO) annotations [11], specific to the gene expression data context, and not useful for any other context. Allied to the fact that the application of biclustering algorithms has not progressed at the same pace as the software development [9], this scenario leads to a collection of biclustering algorithms, whose potential has not been fully explored. The purpose of this study is to evaluate the potentialities of biclustering algorithms in fMRI time series.

Previous exploratory studies considering neurosciences and biclustering did biclustering on extracted features [12–15]. For biclustering, the dimensions were the analysis is done is particularly important, and in contrast to these studies, we do biclustering under the **Region** × **Time** dimensions. We propose that biclustering can be used to analyze the temporal behaviour of brain (divided into a number of regions), and extract correlation regions. The validity of this approach is suggested by a number of studies who applied biclustering to analyse data with spatial and temporal properties [16–28].

Another difference between our work and previous studies is that we do a comparative analysis of the capacities of biclustering fMRI time series considering state-of-the-art biclustering algorithms instead of testing a proposed new algorithm [14, 16, 29]. Therefore, our study resembles biclustering comparison surveys [11, 30–33] but in contrast to them, it uses internal evaluating metrics, which have wider applicability.

We selected seven state-of-the-art biclustering algorithms (covering different search strategies) and applied them to artificial and real-world fMRI datasets. Additionally, we added to this comparison two variations of three popular clustering algorithms, k-means, spectral, and ward’s hierarchical method. In the absence of ground truth to evaluate biclustering/clustering solutions, we opted for internal evaluation metrics and used them to determine the type of patterns to expect in fMRI data.

This study is a comparative study on the application of biclustering in a different biomedical domain than gene expression data analysis. In addition, it proposes a methodology to evaluate biclustering. Besides neurosciences, we suggest that climate science, epidemiology, and sociology, sharing fundamental properties with the former, are scientific fields expected to benefit from this study.

The remaining of this section targets biclustering and related work. First, we position biclustering in the family of unsupervised learning algorithms, describe the relevant biclustering patterns found in real-valued matrices, and categorize biclustering algorithms based on their search strategy. Then we revise previous work on biclustering reviews. Second, we motivate the use of biclustering and review studies about the use of biclustering in neurosciences and spatio-temporal analysis.

The remaining of this paper is organized as follows: In **Methods**, we describe the general comparison methodology followed in our study and how it is different from other comparison studies. In **Results** we introduce and discuss our results. In **Discussion**, we show implications of our study in neurosciences. In **Conclusion**, we give concluding remarks.

### Biclustering

Biclustering can be defined as simultaneous clustering of rows and columns [7], allowing the discovery of rows with similar behavior across a subset of columns, and vice-versa (biclusters). Considering a data matrix *A*, with *n* rows ($X=\{x_1,\ldots ,x_n\}$) and *p* columns ($Y = \{y_1,\ldots ,y_p\}$), the biclustering task aims to discover subsets *B* of the original data matrix *A*. Each subset *B* is called a bicluster, and consists of a subset of rows *I*, and a subset of columns *J*, where $I \subseteq X$ and $J \subseteq Y$.

#### Bicluster patterns

Biclustering is able to find more flexible structures than traditional clustering. While the nomenclature for this possible structures is not standardized in the literature, the definitions provided by Madeira and Oliveira [7] and Aguilar-Ruiz [34] are commonly used. The most simple structure is the **constant bicluster**, which is a submatrix (I,J) where all values $b_{ij}$ are equal: $b_{ij} = \pi$.

In the context of a real-valued matrix, it is of particular interest to discover coherent biclusters. A coherent bicluster uses a row parameter $\pi _i$ and a column one $\beta _j$. The interaction between the two parameters can be either additive (shifting) or multiplicative (scaling). A perfect **shifting bicluster** follows the additive relation among the variable: $b_{ij} = \pi _i + \beta _j$. A **scaling bicluster** follows a multiplicative relation: $b_{ij} = \pi _i \times \beta _j$.

#### Search strategies

When categorizing biclustering algorithms, a particular concern is related to the search strategies. Table 1 systematizes four main search strategies, defined by Madeira and Oliveira [7] and used in recent comparison studies, such as Padilha and Campello [11] and Henriques et al. [33].

**Table 1:** Biclustering search strategies as defined by Madeira and Oliveira [7]

Category	General characteristics	Examples
Greedy	Biclusters are generated by adding or removing columns to a initial random bicluster in order to improve some gain function. The final objective is for the algorithm to find a global minimun solution after some iterations. Despite making wrong decisions, and loosing good biclusters due to beeing stuck in local minima, they have the potential of being fast algorithms	ISA XMotifs [35, 36]
Distribution parameter identification	Assume some statistical model behind the data, and then apply some iterative procedure in order to obtain its parameters by minimizing some criterion	FABIA spectral biclustering [37, 38]
Divide and conquer	Divide the original data matrix into smaller instances. With the potential of being very fast, they could fail to find good biclusters, splitted before identified	Bimax [30]
Exhaustive	Based on the premise that finding the best biclusters can only be done by using an exhaustive enumeration of all possible biclusters in the data matrix. Despite being able to find the bests biclusters they do it by imposing restrictions to the biclusters size (since these algorithms are typically very slow)	BicPAM CCC [39, 40]

#### Reviews

During the last two decades several authors reviewed biclustering methods [6, 7, 9–11, 13, 30–33, 41–44]. These reviews can be fitted into three main categories: (1) General Surveys [6, 7, 9, 10, 13, 33, 44], (2) Comparison Surveys [11, 30–33] and (3) Measure Surveys [41–43, 45].

**General survey** studies categorize biclustering, providing abstract categorizations and reviewing state-of-the-art approaches and typical application cases. These studies are beneficial as a the first look into biclustering.

**Comparison surveys**, approach the task of finding the “best” biclustering algorithm. Prelić et al. [30] was the first to compare multiple biclustering algorithms by adapting methodologies used in clustering. They choose five biclustering methods, proposed a new algorithm, and compared their performance using synthetic and real gene expression data. The validation was performed using external indices on synthetic data and biological relevance. Bozdaǧ et al. [31] compared 5 biclustering algorithms following the approach of Prelić et al. [30], but considering the effects of noise, size and bicluster overlap. Eren et al. [32] expanded the research by comparing 12 algorithms, using synthetic datasets following six different biclustering models and eight gene expression datasets. Henriques et al. [33] compared the performance of pattern-based biclustering to the traditional approaches, evaluating a total of 15 state-of-the-art approaches. Their evaluation was done in synthetic and real datasets, considering two external measures and computational efficiency together with biological relevance of results. Padilha and Campello [11] used three synthetic data collections and two real data collections to analyze the performance of 17 algorithms.

Finally, **measure surveys** discuss metrics to evaluate biclustering.

### Motivating biclustering

The traditional application area for biclustering is in the context of gene expression data analysis. Therefore the number of applications of biclustering in neuroimaging data is limited. This section reviews the most closely related studies. Despite the lack of a research line using biclustering and fMRI data, there are hints in the literature that suggest opportunities for these algorithms to be applied in these data.

**Fig. 1:**
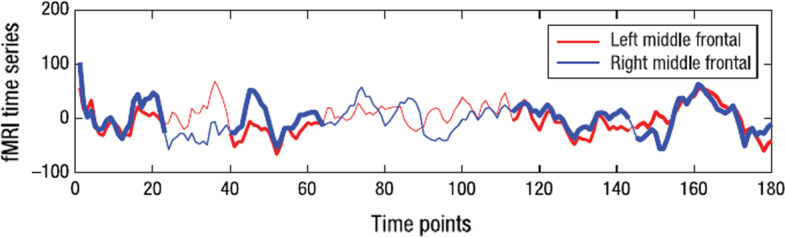
Correlation time series between two non-adjacent brain regions. Biclustering would be able to detect precisely these types of correlation patterns, while ignoring the non-correlated regions and time points. This allow to obtain more flexible structure than traditional clustering (Figure adapted from [1])

**Fig. 2:**
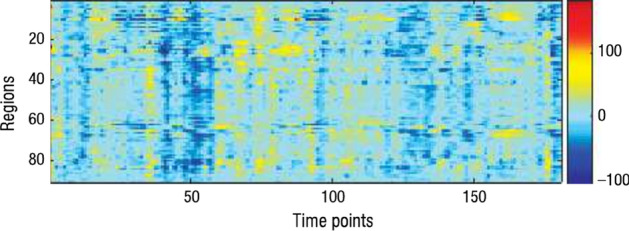
Whole brain time series. In the heatmap, some row and column clusters are visible, as well with some events that happen only for some regions for some specific time points. This later type of structures are not detected by traditional clustering tasks (Figure adapted from [1])

Since the brain structure is dynamic, different brain regions will fire together based on different stimuli [1, 5]. A flexible approach is needed to identify brain regions that fire together under different time points, and can be delivered by biclustering. Figure 1 shows the time-series signal from two non-adjacent brain regions, showing that the correlation between two regions is not static and shows only in some specific time points. Figure 2 shows a whole-brain time-series heatmap, where biclusters-like structures are visible. The biclustering task promises to identify these brain regions interacting together over the time points.

The remaining of this section highlights related studies where biclustering was used. In the first part of this section, we review the various studies that associated neurosciences and biclustering, not necessarily the same type of data as the one we cover. Then, we explain how biclustering is used to analyze data with spatio-temporal properties. While these study cases are outside medical data, they motive the capacities of these methods on temporal data.

#### Biclustering in neurosciences

Busygin et al. [12] used a form of biclustering known as consistent biclustering to analyze EEG data and identify a physiologic marker for optimal vagus nerve stimulator parameters. They conducted biclustering under the **Label** × **Features** dimensions. The labels were created considering experimental settings, and the features are associated with the EEG channels. Fan et al. [13] cited this EEG study as an example of biclustering application, but it is a general-purpose review about biclustering.

Considering structural MRI data, Rahaman et al. [14] proposed a new biclustering approach under the **Subject** × **Features**. In this study, the features are computed considering the application of independent component analysis (ICA) of the MRI spatial maps. Gupta et al. [29] proposed a new biclustering approach in the **Subject** × **Voxel** dimensions to analyse structural MRI data, using an intermediate ICA step.

Yu et al. [15] conducted biclustering analysis to explore relations among brain functional network connectivity (FNC), obtained by using fMRI data and genetic nodes (SNP). Therefore, the biclusters had the **FNC** × **SNP** shape.

In biclustering, the dimensions of analysis are a important part of the analysis. Each one of the previous studies use different dimensions in their analysis, and a different set of dimensions than we do in this study. The study most closely related to our research is the one conducted by Amar et al. [16], that proposed a new algorithm based on biclustering to perform three-way analysis (triclustering). As an application example, they used multi-subject fMRI data (**Region** × **Time** × **Subject**) as an illustrative application of their approach. Their results suggest the capacity of this family of algorithms to identify pertinent brain regions. However, this study is only explorative, meaning that this algorithm’s performance is not tested against other three-way clustering algorithms or approaches.

#### Biclustering spatio-temporal data

In more recent years, more general approaches than clustering have been successfully used in distinct Spatio-temporal application domains. This section focus on the uses of biclustering and triclustering [46] to analyze data with spatio-temporal properties.

Considering biclustering, Wu et al. [17] analysed Chinese meteorological Spatio-temporal data. Shen et al. [18] used biclustering to analyze the global distribution of natural disasters. Kaban et al. [19] used biclustering to identify spatial of social vulnerability in Indonesia and was able to distinguish profiles of social vulnerability. Borgnat et al. [20] identified spatial and temporal profiles in a bike-sharing system in Lyon. Izenman et al. [21] used biclustering to group juvenile-offense data. Dhamodharavadhani and Rathipriya analysed COVID-19 epidemiological data and identified countries with similar epidemic trajectory patterns [27]. Neves et al. [28] used a biclustering algorithm to analyse traffic data.

Considering triclustering, Martínez-Álvarez et al. [22] analyzed parameters to seismogenic zoning using triclustering in the Iberian Peninsula. Guigourès et al. [23] proposed a technique to analyze time-varying graphs, illustrating it in the London bike-sharing system. Wu et al. [24, 25] used triclustering to analyze meteorological data from Duch weather stations. Melgar-García et al. [26] applied a triclustering-based algorithm to discover patterns over time in maize crops in Portugal to help farmers improve their harvests.

It is also relevant to refer that several biclustering algorithms were developed with a focus on temporal data [40, 47–49].

## Methods

**Fig. 3:**
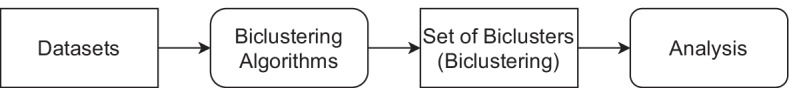
General methodology followed by comparison studies

Comparisons surveys are a category of studies focused on evaluating the perfomance of biclustering algorithms [11, 30–33]. These studies begin by selecting a set of biclustering algorithms and evaluate their perfomance on some dataset. These studies follow a common general strategy that we sistematize in Fig. 3.

In this section, we explain how our study follows this general methodology. For each sub-task, we explain our criteria for each decision and how it distinguishes from other comparison surveys in biclustering [11, 30–33].

### Datasets

Previous studies use two types of data collections: synthetic datasets, where ground truth is known, and real data collections [11, 30–33]. The first is used so that an external metric can be used to evaluate the capacity of the biclustering algorithms to retrieve the artificially-planted biclusters in the dataset [41]. Since data is synthetic, the data parameters can be controlled (such as data size, presence of noise, number of planted biclusters, and possibility of overlapping). The real data collections consists of gene expression datasets from various benchmarks. Typically, the number of datasets in each comparative study is around a few dozens gene expression datasets.

This is a sub-task where our study differ most from previous studies. In our study we work exclusively with fMRI time series datasets, where each dataset represents a brain scan (we use the dimensions **Region** $\times$ **Time**). We use real data and artificial fMRI scans. In our study we use a total of 42 datasets, organized as four data collections, summarized in Table 2.

**Table 2:** Data collections used to evaluate the performance of the biclustering algorithms. We have a total of 42 datasets, each one representing one brain scan

Data collection name	Nature	#Brain scans	#Time points	# Regions	References
“First data collection”	Artificial	1	150	$\approx 30$	[50, 51]
“Artificial data”	Artificial	20	150	$\approx 30$	[50, 51]
“Real data”	Real	20	94	463	[16, 52]
“Illustrative data”	Real	1	137	45	[53–55]

The **First data collection** has a single artificial brain scan, and was used to test biclustering parameters. The **Artificial data** consists of 20 artificial brain scans, which we use to evaluate the performance of the biclustering algorithms. The artificial datasets were obtained using the SimTB simulator [50] to generate data consistent with an auditory oddball experiment [56]. This approach was validated in previous studies and is part of the SimTB software [50, 51]. The **Real data** consits of 20 brain scans, and is also used to evaluate the performance of the biclustering algorithms. The data was collected by Vaisvaser et al. [52] to analyze stress response. This data was preprocessed by Amar et al. [16].^1^ The **Illustrative data** corresponds of a single brain scan, extracted from the “NYU Slow Flanker” dataset [53–55] and is used exclusivelly to illustrate results from a biclustering algorithm.^2^ Typical preprocessing was conducted using the FSL software [57–59]. Finally, the fMRI brain scan was downsampled into regions considering the Harvard-Oxford cortical atlas [60].

### Biclustering algorithms

Dozens of biclustering algorithms have been developed during the last two decades [9]. Since it is not viable to test every single available biclustering algorithm, decisions regarding the best algorithm to use must be made. There is not a single strategy to decide what algorithms to test.

Prelić et al. [30] considered three criteria to chose biclustering algorithms: (1) the popularity of the algorithms in the biclustering community, (2) considering similar algorithmic strategies to be better comparable, and (3) the availability of the implementation. Eren et al. [32] choose algorithms considering both the convenience criteria (algorithms that have available implementations) and the criteria of having various biclustering algorithms with differing approaches. Henriques et al. [33] selected what they considered to be the state-of-the-art biclustering algorithms. Padilha and Campello [11] referred the popularity criteria and the availability of implementation when selecting algorithms.

For our study we considered the following criteria to select biclustering algorithms for analysis:

- We began by considering only popular, freely available implementations of biclustering algorithms. We analysed both the previous comparative studies and the studies considering biclustering time series [11, 30–33, 61].
- We considered important for our algorithms to detect shifting and scaling patterns (patterns commonly found on real-valued datasets).
- The algorithms should be robust to noisy datasets (since there is a lot of expected noise in fMRI time series data).
- The algorithms should cover different search strategies.

We selected seven state-of-the-art biclustering algorithms covering four search strategies. Table 3 highlights general characteristics of these algorithms together with the reasons for their selection.

**Table 3:** Biclustering algorithms considered for this study. Additionally, they will be compared to three popular clustering algorithms: k-means, spectral, and ward’s hierarchical methods. For clustering, we use scikit-learn implementations [64]

Algorithm	Type of search	Available at	References	Reason to choose it
BicPAM	Exhaustive	BicPAMS	[39, 62]	State-of-the-art pattern mining based biclustering method
CCC	Exhaustive	BiGGEsTS	[40, 61]	Allows to obtain temporal contiguous biclusters efficiently
ISA	Greedy	isa2	[35]	State-of-the-art greedy algorithm able to deal with real data
XMotifs	Greedy	biclust	[36, 63]	State-of-the-art greedy algorithm based on a strategy of discretizating data
Bimax	Divide and conquer	biclust	[30, 63]	Very fast algorithm able to detect simple structures
FABIA	Distribution parameter identification	FABIA	[38]	State-of-the-art algorithm
Spectral Biclustering	Distribution parameter identification	biclust	[37, 63]	State-of-the-art algorithm able to detect a specific type of bicluster structures

Since clustering can be viewed as a particular case of biclustering, we can compare biclustering approaches to traditional clustering. Clustering approaches are typically used to group brain-regions with similar activity over time [3, 65–67]. Among the most popular clustering approaches are the K-means algorithm, Spectral Clustering, and Hierarchical methods (in particular using the **Ward’s algorithm**) [66]. However, both row and column clustering make sense and can be considered. In what follows, and considering data format, traditional row clustering and column clustering will be referred as **region clustering** and **temporal clustering**, respectively.

### Biclustering

After running a biclustering algorithm over a dataset, a set of biclusters (biclustering) is generated. Previous studies do not discuss much of this step since it is closely related to the biclustering parametrization step. However, the biclusters must have a format adequate for the posterior analysis.

**Fig. 4:**
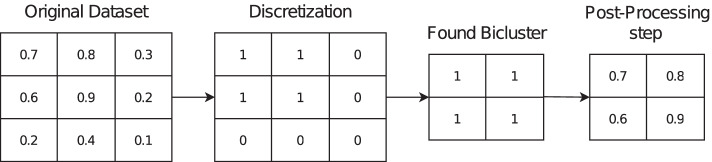
Typically, biclustering algorithms require some preprocessing step (either a normalization or a discretization step). For a fair comparison between multiple biclustering algorithms, a post-processing is done to garantee that the bicluster has the original values present in the original data matrix

Different biclustering algorithms will provide biclusterings with different structures. For example, Bimax requires a binary discretization of the original dataset. Therefore, the output of the algorithm will be a binary bicluster. Our strategy was to consider the set of rows and columns obtained by the algorithm and the original values present in the dataset to define the biclusters to be evaluated. Figure 4 illustrates this process.

Some biclustering algorithms can generate biclusters with less than two rows or columns. In the context of our study, these biclusters were considered uninteresting and were removed from the biclustering, either by parametrization or considering a post-processing step.

For the artificial and real data collections (20 datasets each), biclustering was conducted independently for each dataset. For this study purposes, we consider the union of all generated biclusters as the result for that data collection.

### Analysis

Traditional comparative studies use both the external evaluation metrics applied to the synthetic data and the biological significance applied to the real-world gene expression datasets [11, 30–33].

Our data focus on fMRI time series. External measures cannot be used due to the absence of ground truth. Furthermore, biological significance measures are exclusive to the context of gene expression data and not useful for any other context. Therefore our analysis methodology must be done differently. Internal evaluation indexes were selected to evaluate the performance of biclustering algorithms [43].

Our first task is related to the parametrization of the algorithms. For most parameters, we used the default ones provided by the implementations or suggested by the authors. We decided to test different parameters and select the best performance configurations. This approach is consistent with the standard practice followed by previous studies, which use mostly default parameters, discussing only the effects of some parameters [11, 30, 32, 33]. We use these optimized parameters during the remaining of the study. For this part, the first data collection was used.

Then, we conduct a comparison between biclustering algorithms. This comparison is an essential part of our study since it compares the biclustering capacity against traditional clustering and the capacity of each algorithm. Additionally, we analyze the dimensions of the biclusterings, which are distinct between algorithms and can explain the reasons for the previous performance. Finally, we use the correlation between our metrics to hypothesize the patterns hidden in fMRI time series. For this analysis, we used the artificial data collection and the real data collection.

#### Evaluation metrics

In our study, we use internal evaluation indexes, that translate the internal quality of the bicluster into a single number [43]. However, since there are multiple types of patterns that fall into the definition of bicluster, there is no single metric that can capture all types of patterns. As stated by Hartigan [68], one simple hypothesis to evaluate a bicluster is to calculate its **Variance (VAR)**:

1 $$\begin{aligned} VAR(B) = \frac{1}{\mid I \mid \times \mid J \mid } \sum _{i=1}^{\mid I \mid }{\sum _{j=1}^{\mid J \mid }{(b_{ij} - b_{IJ})^2}}, \end{aligned}$$

where $b_{ij}$ refers to the element (*i*, *j*) of the bicluster *B*, $b_{IJ}$ refers to the global mean of the elements belonging to the data matrix and |*I*|, |*J*| represent the total number of rows and columns of the bicluster. A disadvantage of using the variance is that it is only able to measure constant biclusters. Therefore it will fail to detect most of the more complex patterns we want to detect.

Introduced by Cheng and Church [8], the **Mean Squared Residue (MSR)** aims to find the coherence of the bicluster over rows and columns:

2 $$\begin{aligned} MSR(B) = \frac{1}{\mid I \mid \times \mid J \mid }\sum _{i=1}^{\mid I \mid }{\sum _{j=1}^{\mid J \mid }{(b_{ij} - b_{iJ} - b_{Ij} + b_{IJ})^2}}, \end{aligned}$$

where $b_{iJ}$ and $b_{Ij}$ point to the row *i* mean and the column *j* mean. This approach is an improvement compared to the variance since it can capture shifting tendencies. However, it is unable to capture scaling patterns [31].

In order to capture the scaling tendencies that the MSR cannot recognize, Mukhopadhyay et al. [69] developed a new measure called **Scaling Mean Squared Residue (SMSR)**, focused on scaling patterns (however, it fails to capture the shifting patterns).

3 $$\begin{aligned} SMSR(B) =\frac{1}{\mid I \mid \times \mid J \mid }\sum _{i=1}^{\mid I \mid }{\sum _{j=1}^{\mid J \mid }{\frac{(b_{iJ} \times b_{Ij} - b_{ij} \times b_{IJ})^2}{b_{iJ}^2 \times b_{Ij}^2}}}. \end{aligned}$$

An important property of biclusters is the possibility that they could have a different range of values between each other, suggesting the use of standardisation processes. As pointed by Pontes et al. [43], an advantage of this approach is to characterise their tendency. One way of doing it is to standardize data by row. In the context of fMRI, this means normalizing each brain region to have a similar amplitude as follows:

4 $$\begin{aligned} {\hat{b}}_{ij} = \frac{b_{ij}-\mu _{g_i}}{\sigma _{g_i}}, \quad 1 \le i \le \mid I \mid , 1 \le j \le \mid J \mid , \end{aligned}$$

where $\mu _{g_i}$ and $\sigma _{g_i}$ represent the mean and standard deviation over the rows of the bicluster. Based on this approach, a quality measure was proposed under the name of **Virtual Error (VE)** [34, 70], considering the concept of a virtual pattern, which can be defined over rows or columns [43]. The normalized pattern over rows is given by:

5 $$\begin{aligned} {\hat{\rho }}_{j} = \frac{1}{\mid I \mid } \sum _{i=1}^{\mid I \mid }{{\hat{b}}_{ij}}. \end{aligned}$$

This pattern represents the average brain behaviour over time, and the virtual error is thus defined as a measure of the difference between the real region behaviour compared to this virtual row:

6 $$\begin{aligned} VE(B) = \frac{1}{\mid I \mid \times \mid J \mid }\sum _{i=1}^{\mid I \mid }{\sum _{j=1}^{\mid J \mid }{\mid {\hat{b}}_{ij} - {\hat{\rho }}_{j} \mid }}. \end{aligned}$$

A particular advantage of the virtual error measure is its capacity to detect both shifting and scaling patterns [43]. This advantage means that it is a suitable internal measure to compare different possible patterns obtained by different biclustering methods. When considering the presence of noise, Pontes et al. [43] pointed out that the virtual error can capture both scaling and shifting patterns since its value varies linearly with the induced error. Additionally, this metric is of particular interest since it uses the concept of pattern over a “time” dimension.

#### Types of signal

While the virtual error is expected to capture both shifting and scaling patterns [43], it does not provide any knowledge in ther of the specific type of the signal found in the fMRI data. Padilha and Carvalho [71], in a study consisting of 9 biclustering algorithms and a benchmark of 19 different real gene expression datasets, showed that the virtual error is not fundamentally correlated with any of the remaining discussed measures (variance, MSR and SMSR).

Since the virtual error promises to be a stable measure for different types of biclusters, we expect it to be correlated with the measure able to find the most common pattern present on our data. Therefore, we used the Pearson coefficient between the three mentioned quality measures (VAR, MSR and SMSR) and the virtual error measure to get insights patterns found in our data.

## Results

This section discusses the performance of biclustering algorithms in our evaluation scenarios.

We begin by testing the influence of different parameters on the performance of each algorithm, to select an adequate set of parameters for the algorithms to be compared with each other. Then, we evaluate the perfomance of the biclustering algorithms considering the following criteria: (1) biclustering versus region and temporal clustering; (2) different biclustering algorithms; (3) top-K biclusters; (4) bicluster size; (5) type of bicluster pattern found.

### Testing configurations

In this scenario, the objective was to test the behaviour of different parameter configurations on six bicluster algorithms (BicPAM, FABIA, Bimax, CCC, XMotifs and Spectral). The First data collection was used for this step, and Table 4 shows the results of this testing.

**Table 4:** Median values for the four selected measures for the first artificial dataset, with uncertainties given by the standard deviation (except for the case of SMSR were the standard deviation is orders of magnitude higher than the median value). From these results it is visible that **A)** The high values of uncertainty discourage focus on optimizing the biclustering method parameters and **B)** Choosing the right evaluation metric is important, however in most of the biclusters cases they seem to agree for the same “best” configuration. Bold represents the choosen parameters for the next sections

Method	Configuration	VAR	MSR	SMSR	VE
BicPAM	**Additive version**	$0.024 \pm 0.024$	$0.001 \pm 0.003$	2.49	$\mathbf{0} .\mathbf{262} \pm 0.232$
	Constant version	$0.014 \pm 0.026$	$0.001 \pm 0.002$	1.96	$0.434 \pm 0.185$
	Multiplicative	$0.025 \pm 0.024$	$0.002 \pm 0.005$	3.07	$0.461 \pm 0.263$
Bimax	10 biclusters	$0.004 \pm 0.002$	$0.001 \pm 0.001$	0.03	$0.881 \pm 0.148$
	100 biclusters	$0.006 \pm 0.004$	$0.003 \pm 0.002$	0.05	$0.729 \pm 0.128$
	1000 biclusters	$0.008 \pm 0.008$	$0.004 \pm 0.004$	0.06	$0.706 \pm 0.168$
	**10,000 biclusters**	$0.008 \pm 0.007$	$0.004 \pm 0.004$	0.06	$\mathbf{0} .\mathbf{695} \pm 0.169$
	100,000 Biclusters	$0.008 \pm 0.007$	$0.004 \pm 0.004$	0.06	$0.695 \pm 0.169$
CCC	**Traditional discretization (5 symbols)**	$0.189 \pm 0.359$	$0.017 \pm 0.021$	0.51	$\mathbf{0} .\mathbf{288} \pm 0.345$
	Variation between time points (2 Symbols)	$0.596 \pm 0.491$	$0.067 \pm 0.108$	0.93	$0.370 \pm 0.315$
	Variation between time points (3 Symbols)	$0.596 \pm 0.491$	$0.067 \pm 0.108$	0.93	$0.370 \pm 0.315$
FABIA	**Standard**	$0.579 \pm 0.502$	$0.006 \pm 0.081$	0.51	$\mathbf{0} .\mathbf{079} \pm 0.167$
	Relaxed	$0.946 \pm 0.053$	$0.881 \pm 0.095$	3485.60	$0.787 \pm 0.036$
Spectral Biclustering	**log**	$0.039 \pm 0.053$	$0.033 \pm 0.004$	670.30	$\mathbf{0} .\mathbf{726} \pm 0.012$
	bistochastization	$0.040 \pm 0.005$	$0.035 \pm 0.004$	3232.21	$0.731 \pm 0.015$
	irrc	$0.040 \pm 0.004$	$0.035 \pm 0.004$	1271.71	$0.726 \pm 0.015$
XMotifs	Discretization with 2 symbols	$0.017 \pm 0.004$	$0.012 \pm 0.001$	1168.76	$0.662 \pm 0.048$
	**Discretization with 5 symbols**	$0.007 \pm 0.009$	$0.003 \pm 0.001$	6.59	$\mathbf{0} .\mathbf{585} \pm 0.035$

In terms of input, the format Region × Time was selected to run most of the algorithms. This approach was chosen since the algorithms are in general implemented to run in this configuration, since they were designed in gene expression context, where genes are commonly used in the rows and the conditions, such as time, fit in the columns [7].

Considering the input format, an exception was made for **BicPAM**, since this algorithm was reported to be more efficient for matrices with a larger number of rows than columns (which for this dataset consists of the Time × Region format), and allows to select a dimension to search for patterns (the temporal dimension was chosen) [39, 62]. Due to its flexibility of finding different types of patterns, BicPAM was also run in three different configurations to search for constant, shifting and scaling patterns. The *minimum number of biclusters before merging* parameter was selected as high as possible in order to guarantee an adequate exploration of the dataset, while forcing it to run in a reasonable time. Additionally, BicPAM has a discretization step, and we used the default five symbols discretization. Based on the virtual error results between constant, shifting and scaling patterns, we choose the “additive” version of BicPAM.

In **FABIA** the number of biclusters is always limited: it cannot be higher than the number of rows and the number of columns. In this case, we selected the number of columns of the dataset as a number of datasets (following the methodology proposed by Henriques et al. [33]). We considered two threeshold options: (1) default thresholds, and (2) relaxed values to try to find more biclusters. This treeshold influences the size and number of generated biclusters.

We observed that the relaxed configuration achieves far worse results than the standard configuration. This suggests that the strategy of relaxing the thresholds does not provide more meaningful results, therefore we used the default threshold values.

In **Bimax** the default values were used but for the number of biclusters, changed as follows: 10 biclusters, 100 biclusters, 1000 biclusters, 10,000 biclusters and 100,000 biclusters.

We observed that generating more biclusters mean adding more noise to the previous generated ones. Additionally, we observed an effect of saturation when generating a large number of biclusters, since asking the algorithm to generate “100,000” biclusters does not produce actually any more biclusters than the “10,000” option.

The greedy algorithm **XMotifs** was executed 30 times (to avoid interference from the starting seeds, and following the approach proposed by Padilha and Campello [11]). XMotifs uses a discretization step, and 5 symbols were used (in order to be similar to the discretizations used by CCC and BicPAM), as well as 2 symbols (to be similar to Bimax). The strategy of using a discretization with 5 symbols provided biclusters with smaller errors than using a binary discretization. Similar results were obtained by Kemal et al. [32].

**Spectral biclustering** uses a normalization method. We used the three normalization methods made available by the authors: “logarithmic normalization” (log), “independent rescaling of rows and columns” (Irrc) and “bistochastization” [37]. The three configurations generate similar results in all metrics (except SMSR). The “log” configurations achieved the best performance in all four metrics, thus since it is also the configuration recommended by the authors, it was selected for the next stages of research.

In **CCC** most of the parameters are associated with the discretization step. We used the two major possibilities: use a traditional discretization per row (for this, 5 symbols was used) or use variation between time points (2 or 3 symbols) as described in Madeira et al. [40]. A surprising result is the traditional discretization to generate best biclusters than the variations options, contradicting results obtained by Madeira et al. [40]. This could be due to two possible reasons: different application context or the use of a different evaluation metric.

### Comparing biclustering and clustering

**Fig. 5:**
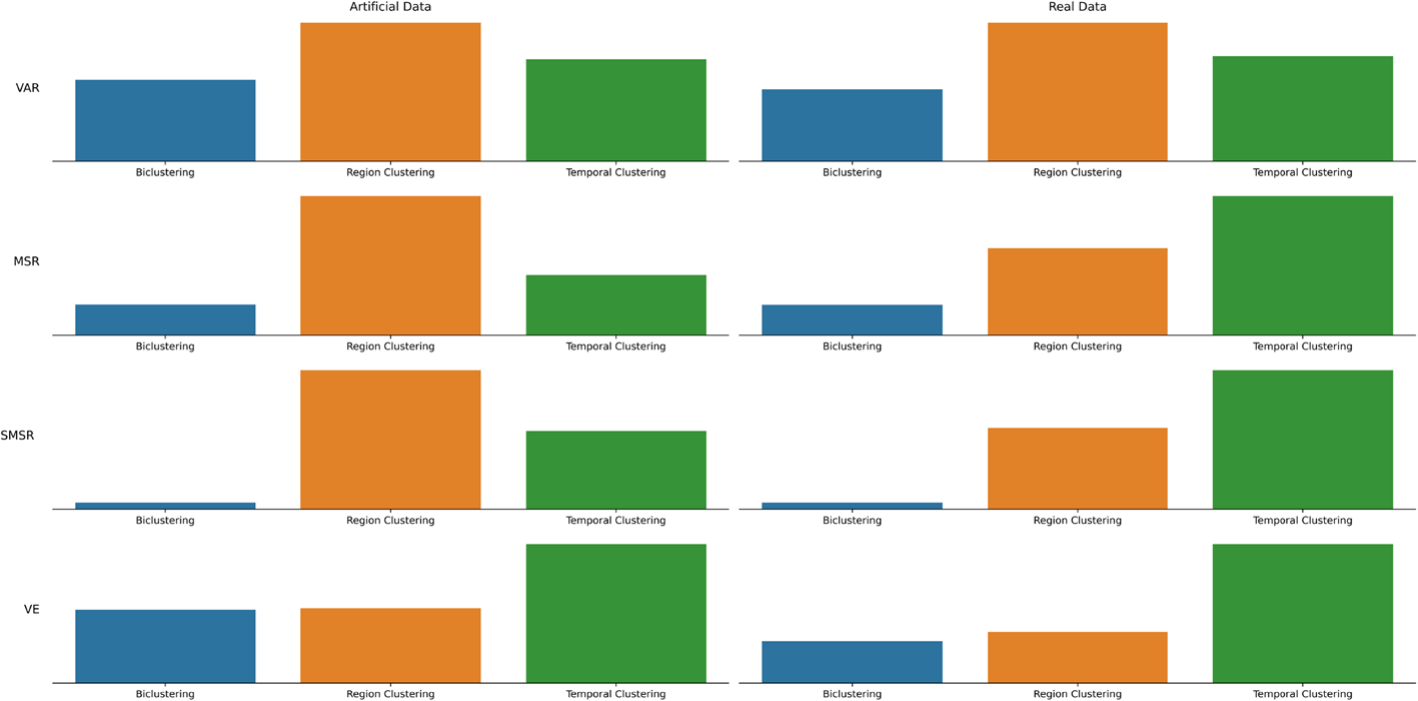
Comparing the general capacity of biclustering algorithms versus the two clustering variants in artificial and real data. This is done by aggregating the results from all algorithms and comparing the median value of the metric. The results motivate the capacity of biclustering to obtain promising results for analysing the data

In this scenario, the general objective is to compare the general capacity of biclustering algorithms with traditional clustering algorithms in both region and temporal clustering. Results are summarized in Fig. 5. The general conclusion anticipates biclustering to obtain more homogeneous structures than the traditional clustering structures, since it achieves better values in all four quality evaluation criteria.

### Comparing algorithms

**Fig. 6:**
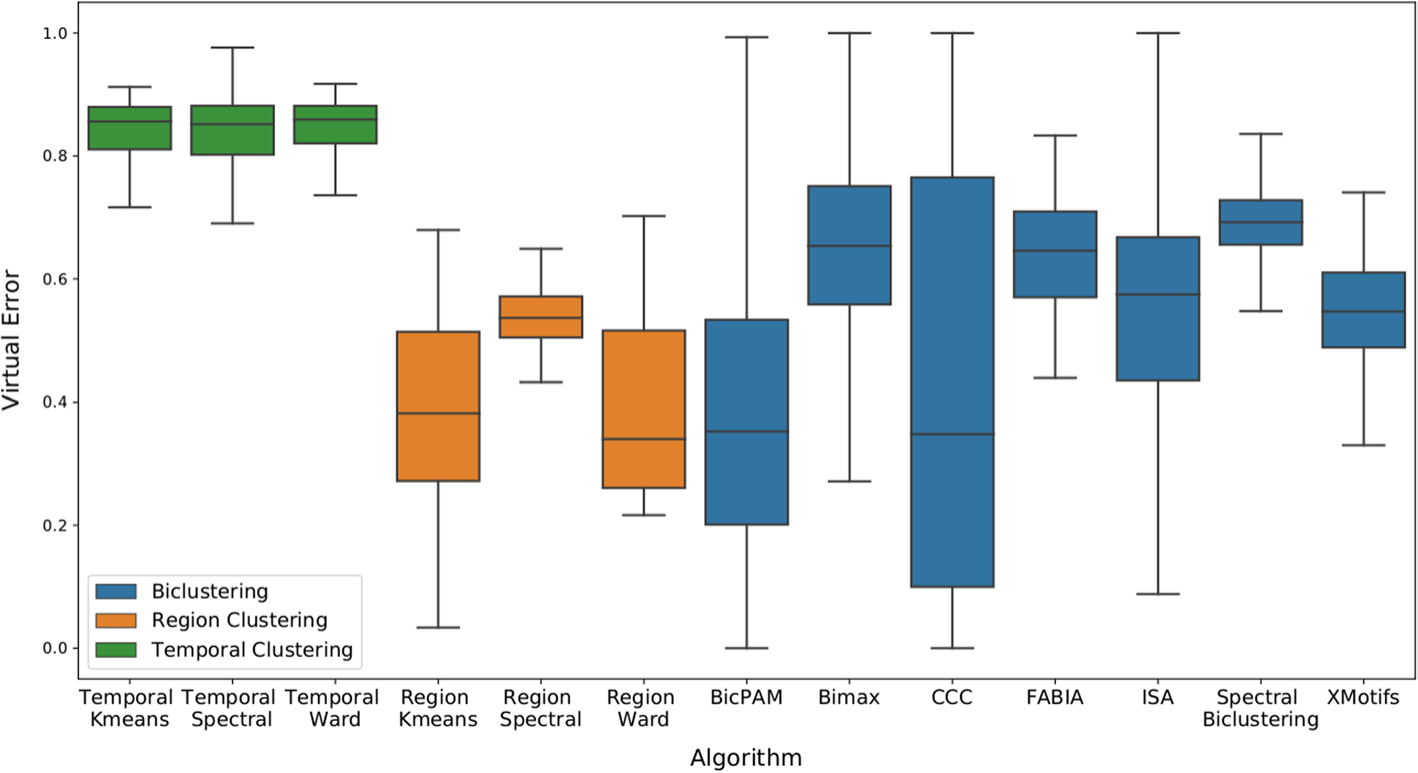
Virtual Error measure for every tested algorithm in our artificial data collection. Despite having great oscillations, the median performance of the exhaustive approaches (CCC and BicPAM) show promising results in comparison with the remaining biclustering approaches

**Fig. 7:**
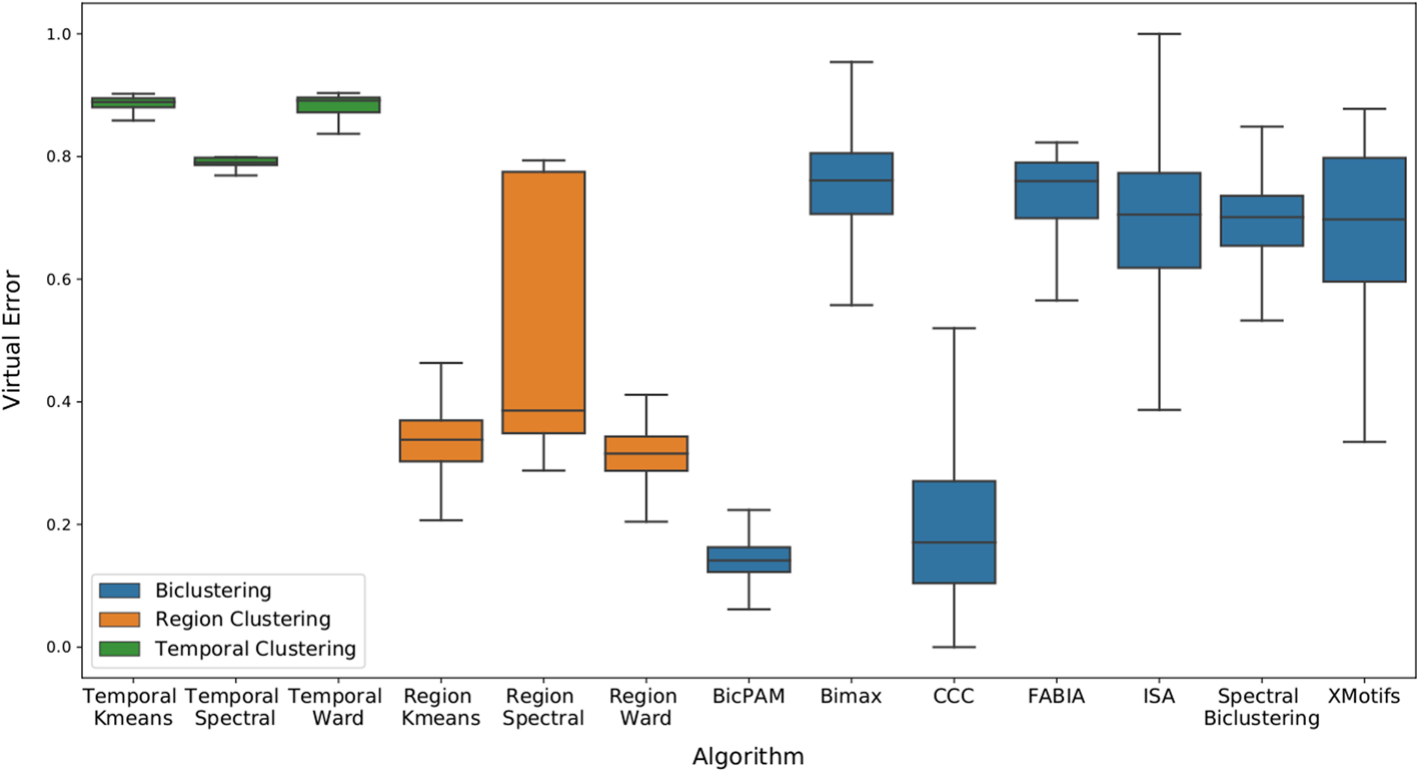
Virtual Error measure for every tested algorithm in our real data collection. Despite the biclustering algorithms not being indisputable better than the traditional clustering, the use of exhaustive biclustering approaches such as CCC and BicPAM show a good capacity of generating coherent biclusters

While previous results motivate the use of biclustering, they do not explain the capacity of each individual algorithm to generate homogeneous biclusters. In this scenario we tested the performance of each biclustering (and clustering) algorithm. Figures 6 and 7 illustrate our results in both synthetic and real data.

The first immediate conclusion is related with the lack of capacity of temporal clustering algorithms to generate good results. This makes sense since these clustering scenarios generate groups of time points where all regions behave similarly. Since the brain is heterogeneous, different regions will behave differently under the same time points, it was not expected that clustering would generate good results considering the virtual error measure.

More comparable are the tasks of region clustering and biclustering. In this scenario, most biclustering algorithms achieve worse results than the traditional clustering tasks, with the exception of exhaustive biclustering algorithms. However, the capacity of the exhaustive approaches (BicPAM and CCC) to find homogeneous biclusters shows that while the clustering methods are fundamentally limited (due to the cluster structure), the biclustering task is able to achieve very good results.

Focusing on the individual performance, it is not too surprising that the Bimax algorithm would achieve bad results since it discretizes data in a very specific way (it searches for binary biclusters). This step of turning the dataset into a binary one strongly limits the capacity of finding good biclusters. For Spectral Biclustering results are not surprising, since it does search for a very specific type of bicluster. FABIA has similar problems when compared to clustering approaches, since its factor-analysis approach strongly limits the capacity of the algorithm of generating biclusters. Both ISA and XMotifs produce bad results, which is suprising since they were expected to find the same types of biclusters than the exhaustive algorithms.

### Comparing top-K biclusters

Biclustering solutions are composed of a different number of generated biclusters, which can be order of magnitude different depeding of the algorithm. Additionally, as part of the methodology, greedy algorithms were executed multiple times to avoid interference from their stochastic nature. Therefore, comparisons that use every single bicluster are not be fair. In this section, our strategy was to use the virtual error as a filter and select the *top-K* biclusters of each algorithm. Additionally, to avoid statistical artifacts, biclusters with virtual error smaller than 0.01 were removed from our study.

**Fig. 8:**
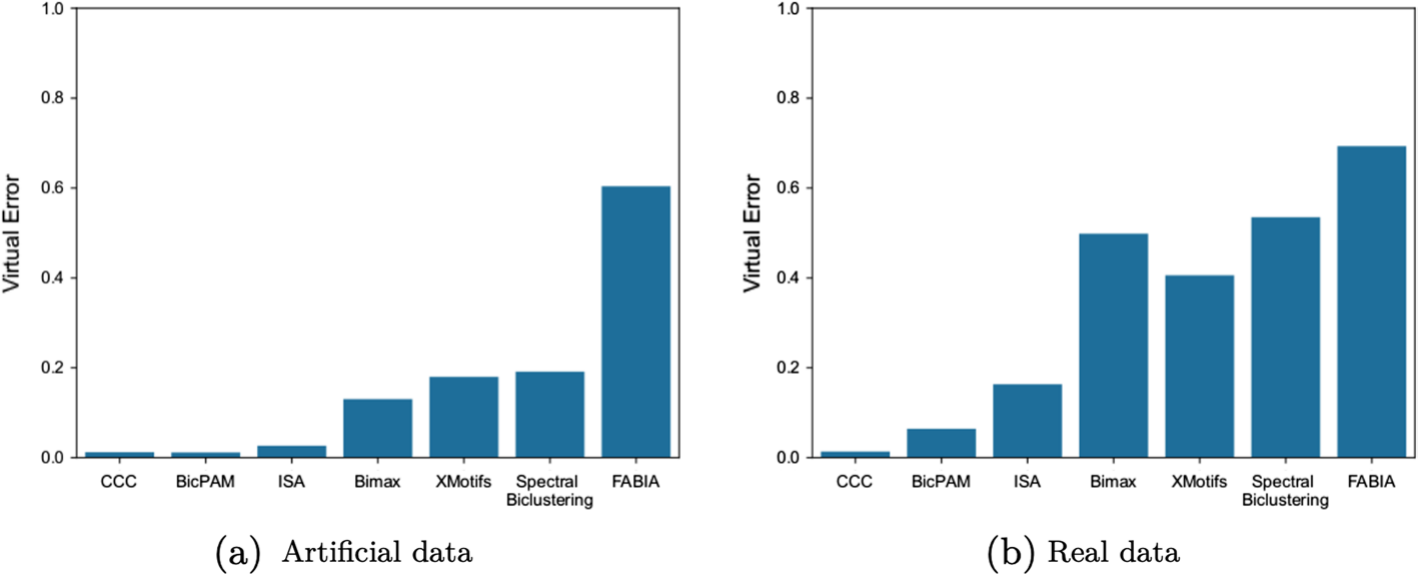
Comparison between the best generated biclusters for each biclustering algorithm. From the previous biclustering solution, the top-k biclusters (filtering by the virtual error and removing biclusters with virtual error smaller than 0.01). The results follow the previous conclusions pointing for a high capacity of the exhaustive algorithms to generate good biclusters. Additionally the ISA results suggest that, while is general performance is bad, it does have the capability of generating some good biclusters

In our study, we selected the *K* value empirically considering the number of already generated biclusters. For the artificial data, the *K* number was selected as 50, and for the real dataset, the selected number was 500. Our results are shown in Fig. 8 and reinforce the capacity of exhaustive approaches to generate homogeneous biclusters. Additionally, ISA had a great improvement in performance, indicating that it could be an interesting choice for dealing with this type of data. XMotifs, while being also a greedy algorithm had a worse performance but still better than the FABIA and Spectral algorithms (that are based on distribution parameter identification).

### Size remarks

In this section, we studied the size of the biclusters to get insights of possible reasons some algorithms achieve better results than others. Since the algorithms generate biclusters with different properties, now we focus on bicluster size parameters. Our results are shown in Table 5.

**Table 5:** Median values (and associated standard deviation) for the typical bicluster dimension parameters in both data collections: number of regions in each bicluster, number of time points and bicluster area. When comparing this results to the virtual error values, a apparent relation comes between the bicluster size and the associated virtual error, which make sense

Algorithms	Artificial data			Real data		
	Time points	Region points	Area	Time points	Region points	Area
*Biclustering*						
Bimax	$6 \pm 7$	$4 \pm 2$	$24 \pm 25$	$6 \pm 2$	$21 \pm 21$	$126 \pm 386$
BicPAM	$7 \pm 3$	$2 \pm 1$	$16 \pm 11$	$27 \pm 8$	$2 \pm 0$	$54 \pm 16$
CCC	$3 \pm 4$	$3 \pm 2$	$10 \pm 9$	$5 \pm 2$	$4 \pm 9$	$18 \pm 22$
FABIA	$43 \pm 5$	$4 \pm 2$	$184 \pm 76$	$29 \pm 4$	$14 \pm 52$	$403 \pm 1204$
Spectral Biclustering	$23 \pm 11$	$7 \pm 3$	$136 \pm 112$	$20 \pm 7$	$17 \pm 6$	$330 \pm 211$
ISA	$14 \pm 9$	$3 \pm 1$	$44 \pm 46$	$7 \pm 5$	$30 \pm 28$	$152 \pm 276$
XMotifs	$49 \pm 13$	$2 \pm 1$	$114 \pm 45$	$19 \pm 11$	$4 \pm 15$	$78 \pm 71$
*Region clustering*						
kmeans	150	$3 \pm 2$	$450 \pm 283$	94	$4 \pm 1$	$376 \pm 123$
pectral	150	$4 \pm 4$	$525 \pm 610$	94	$3 \pm 117$	$282 \pm 11038$
ward	150	$6 \pm 5$	$900 \pm 700$	94	$3 \pm 1$	$282 \pm 100$
*Temporal clustering*						
kmeans	$3 \pm 5$	26	$78 \pm 119$	$3 \pm 0$	463	$1389 \pm 197$
spectral	$3 \pm 6$	26	$78 \pm 158$	$86 \pm 36$	463	$39818 \pm 16621$
ward	$3 \pm 9$	26	$78 \pm 247$	$3 \pm 0$	463	$1389\pm 222$

A first view on the results show biclustering solutions tend to generate biclusters with highly variable sizes. This makes sense, since the search strategies of each algorithm are different.

In the previous sections we observed the capacity of exhaustive algorithms to generate better biclusters than the remaining algorithms. This results show some possible hits of the reasons why they do it, since CCC and BicPAM generate the smallest biclusters in terms of area. For BicPAM these results show a lack of subspace exploration. BicPAM achieves great results by allowing to generate very small biclusters. Additionally, while the algorithm promised to generate biclusters of all sizes (due to its exhaustive nature), this comes at a price of execution speed (which we observed to be far greater than the remaining algorithms) and a running memory price, making this promise to be potentially unfeasible. Despite being also exhaustive, the temporal contiguity constraint of CCC lead to a faster exploration of the datasets.

FABIA produces the largest biclusters. This is related to the search strategy that uses factor analysis as base. For Spectral, the use of singular value decomposition and the generated checkerboard structures help explain the size of the generated biclusters. Bimax generates biclusters with different sizes due to its simplistic approach. The greedy algorithms ISA and XMotif generate relatively small biclusters.

Finally, clustering approaches generate the largest solutions. This is strongly related with their restriction of including all rows (or columns) in the clustering.

### Types of bicluster patterns

To detect the pattern structures found by the biclustering algorithms, we calculated the square of the Pearson coefficient between the VAR, MSR and SMSR and the Virtual Error. A high correlation could be indicative of the expected type of pattern. Our results are shown in Table 6.

Most biclusters agree that the expected patterns are of shifting nature. Bimax is one of the exceptions, supporting constant and scaling patterns. However, it must be pointed that Bimax does not recognize any special type of structure since it works only with binary data. Other exception is CCC, supporting the hypothesis of constant patterns. This could be a consequence of the temporal contiguity constrain that generates biclusters that are fundamentally different than the ones obtained by the other algorithms.

**Table 6:** Correlation between the virtual error and the three specific coherence measures: Variance (constant biclusters), MSR (shifting biclusters) and SMSR (scaling biclusters). Most of the algorithms agree that the expected patterns are of shifting nature

Algorithms	Artificial Data			Real Data			Type of Pattern
	Variance	MSR	SMSR	Variance	MSR	SMSR	
BicPAM	0.009	**0**.**133**	0.000	0.040	**0**.**306**	0.000	Shifting
Bimax	0.003	0.037	**0**.**087**	**0**.**196**	0.001	0.000	Constant/scaling
CCC	**0**.**112**	0.007	0.000	**0**.**200**	0.033	0.000	Constant
FABIA	0.051	**0**.**627**	0.061	0.003	**0**.**763**	0.001	Shifting
ISA	0.050	**0**.**453**	0.000	0.040	**0**.**045**	0.000	Shifting
Spectral biclustering	0.009	**0**.**125**	0.000	0.103	**0**.**361**	0.000	Shifting
XMotifs	0.115	**0**.**378**	0.016	0.003	**0**.**670**	0.000	Shifting

## Discussion

Biclustering is a technique that allows the simultaneous clustering of rows and columns. It is worth noticing that the application of biclustering has not progressed in parallel with algorithm design. This has two particular reasons. The first one happens due to a gap between tool development and the understanding of the data properties for each specific study. The second one is a knowledge gap for applying biclustering with other analytical tools such as annotation processes, visualization programs and statistical methods, to derive a more comprehensive interpretation [9].

In the context of neurosciences, this gap is wider for two reasons: first, previous comparative studies consider only the gene expression context in their evaluations. Second, there is a scarcity of a consistent research line for the interpretation and application of biclustering.

Our study aimed to close this gap between algorithm design, software development and the application of biclustering. The first part of this study was a comparative study for the biclustering capacities of extracting patterns from fMRI time-series data. In this section, we discuss the second issue and highlight the potential of biclustering for fMRI data analysis.

The **first** sub-section is an illustrative analysis of a single fMRI scan, highlighting the spatial and temporal patterns that biclustering can discover in fMRI. The **second** sub-section discusses how biclustering could be integrated with other state-of-the-art techniques of fMRI data analysis.

### Illustrative results

**Fig. 9:**
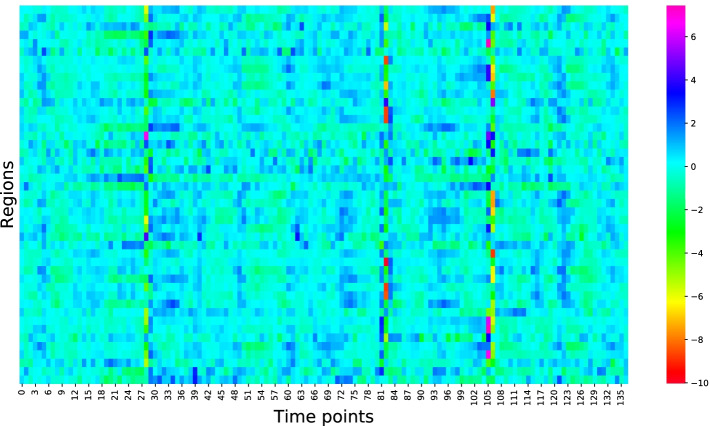
Heatmap of the illustrative data. Interaction between brain regions is local: some brain regions interact together in some time points. Traditional clustering analysis is not able to automatically discover these structures

To highlight the potentialities of biclustering to detect interesting structures, we consider the “Illustrative data” (already mentioned in methods). This dataset consists of an fMRI scan of 137 time points and 45 brain regions (the Harvard-Oxford atlas was used to group the brain in regions). A heatmap of our data is illustrated in Fig. 9, where several bicluster-like structures are visible.

**Fig. 10:**
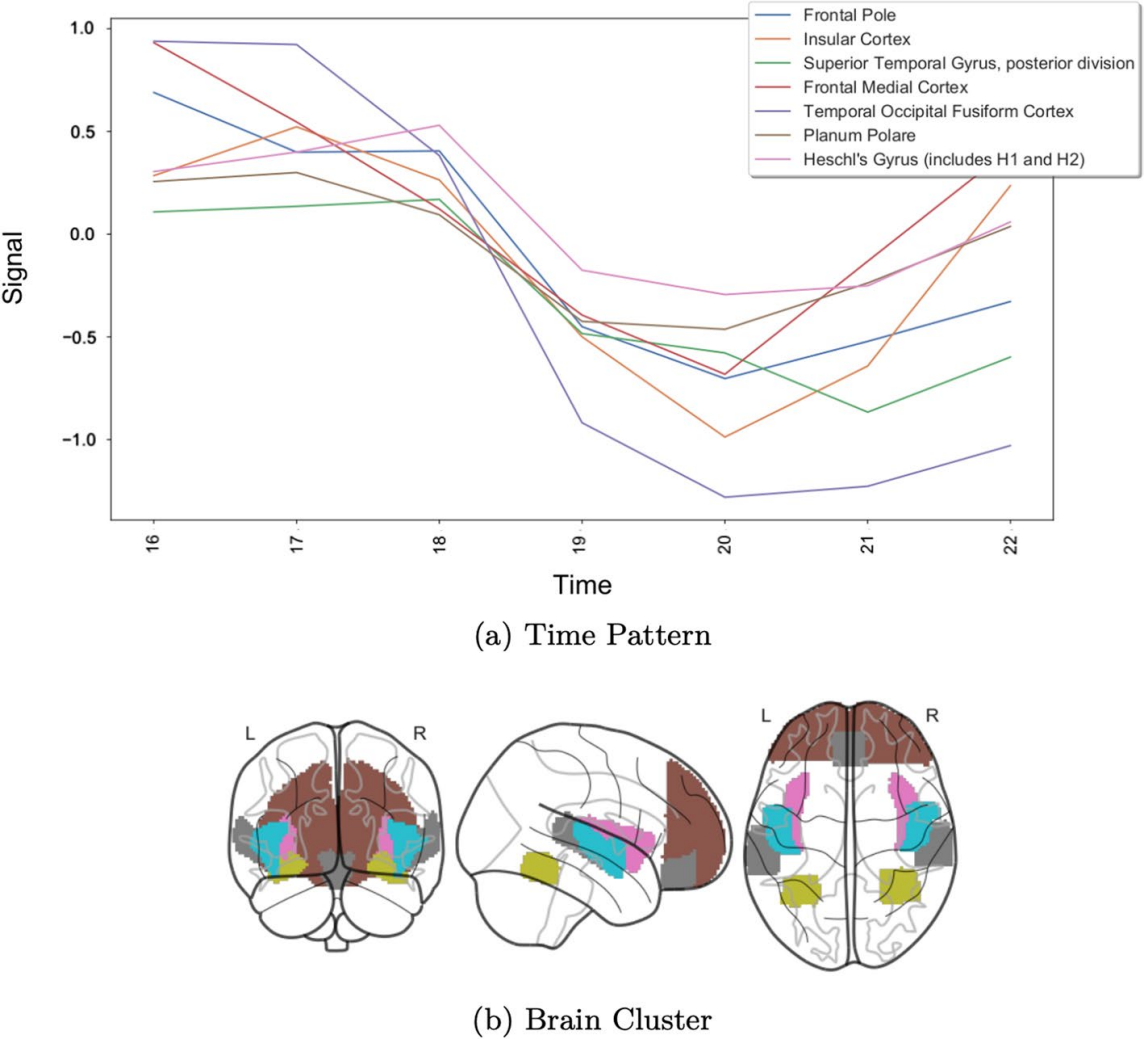
First example bicluster

**Fig. 11:**
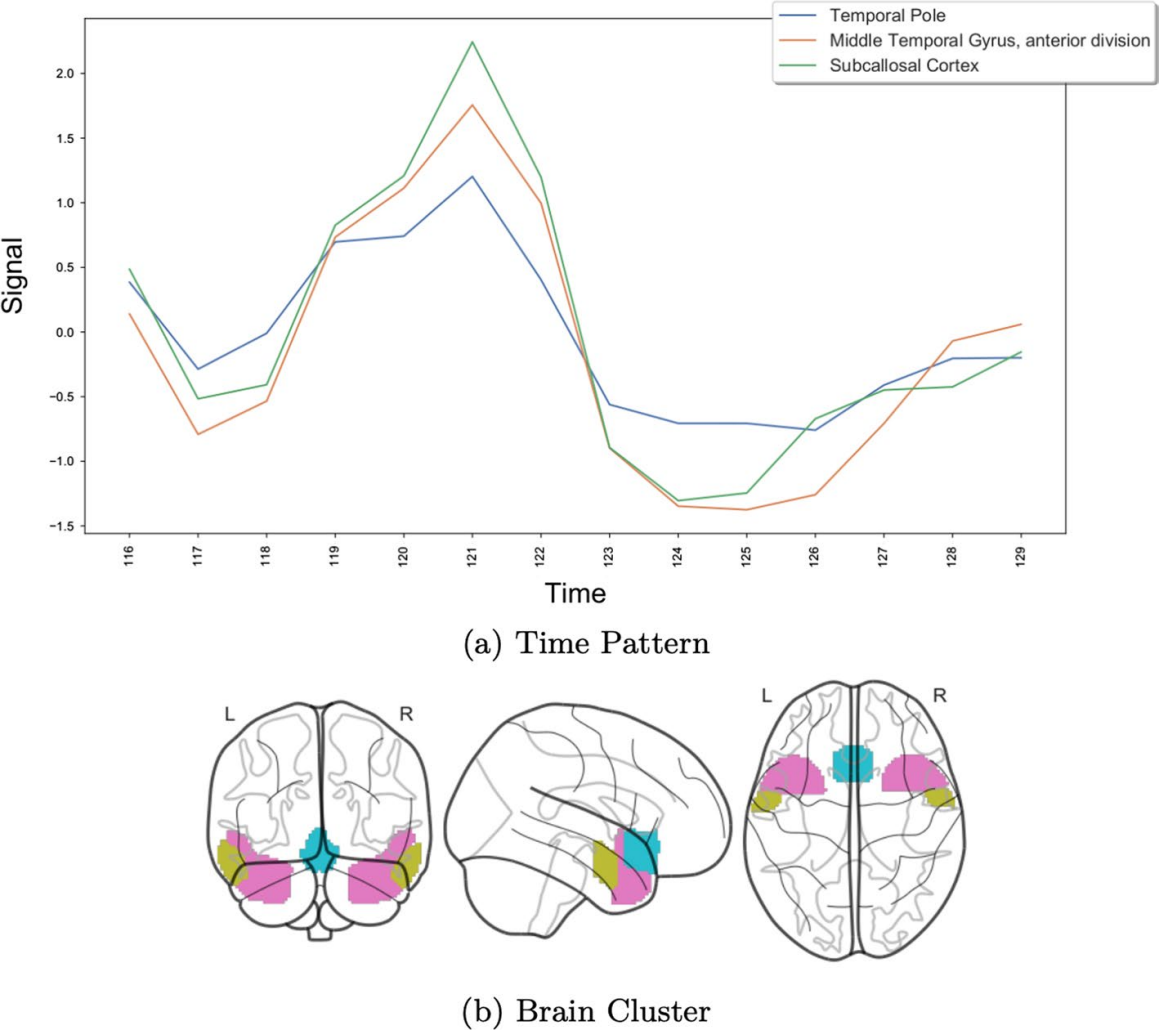
Second example bicluster

**Fig. 12:**
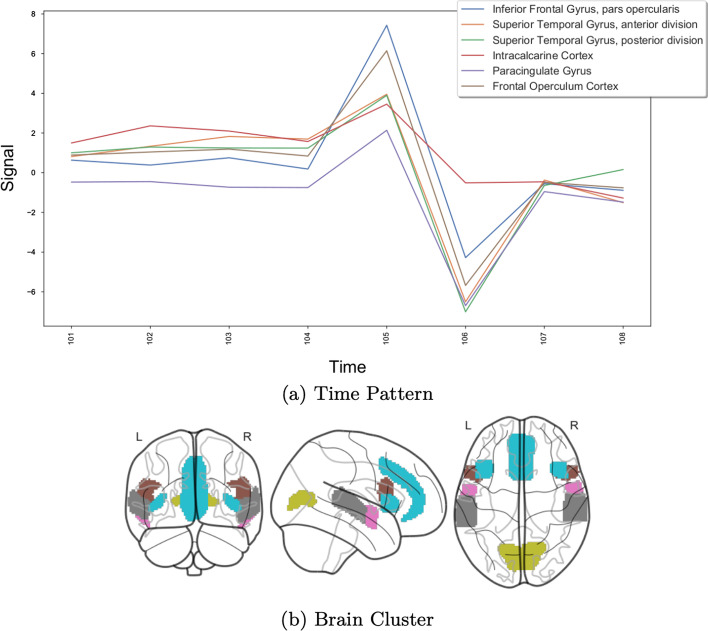
Third example bicluster

We choose the CCC biclustering algorithm, implemented in BiGGEsTS [40, 61]. This algorithm was chosen, not only because it achieved good results in the previous analysis, but to generate temporal contiguous biclusters with an easier interpretation. The algorithm returned 749 biclusters, which we sorted according to a temporal statistical significance metric [40, 45]. Figures 10, 11 and 12 show three of the most relevant biclusters.

### Biclustering opportunities in fMRI data analysis

Biclustering is not a standalone technique, and is often integrated with an analysis pipeline consisting of several other tools, such as results annotation processes, visualization programs and statistical methods [9]. We explore how biclustering could be integrated with other popular fMRI analysis techniques: clustering, independent component analysis and graph theory. In each section, we analyse how biclustering compares to them, and how these techniques can be combined to achieve greater interpretations of the phenomena under study. In addition to these, we explore how biclustering, an unsupervised technique of analysis is used to improve the quality of classification tasks.

#### Clustering

**Fig. 13:**
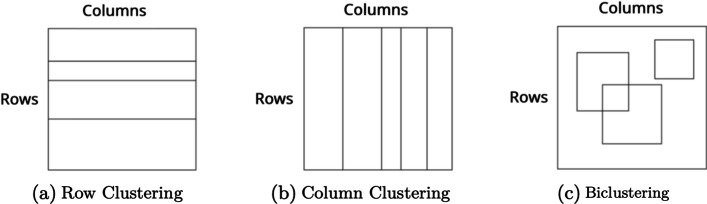
Diferentes between Clustering and Biclustering. While clustering methods allow to obtain only disjoint strips in the data matrix, biclustering finds more flexible structures (Figure adapted from [6])

Biclustering algorithms were originally developed to expand on clustering limitations, in particular to allow overlapping between structures and searching for similarity considering only a subset of the features. Therefore, while clustering allows only to discover disjoint structures (subsets of rows or columns), biclustering discovers a larger set of possible interactions. Figure 13 illustrates differences between clustering and biclustering.

If directly applied to fMRI time series (in the format **Region** $\times$ **Time**), clustering discovers subsets of regions that have a similar behaviour considering *all* datasets. This approach assumes that all brain regions behave statically in time. Biclustering is expected to overcome this assumption and search for local patterns.

#### Indepedent component analysis

Indepedent Component Analysis (ICA) is a dimensionality reduction technique that separates a multivariate signal into a number of components [72]. Often used for exploratory data analysis, in fMRI is particular popular since it separates the analysis of a **Time** × **Region** dataset into their temporal (**Time** × **components**) and spatial (**components** $\times$ **Space**) parts [73]. ICA is used in fMRI to analyse static brain connectivity and has been used to consistent networks across several studies [74–77].

**Fig. 14:**
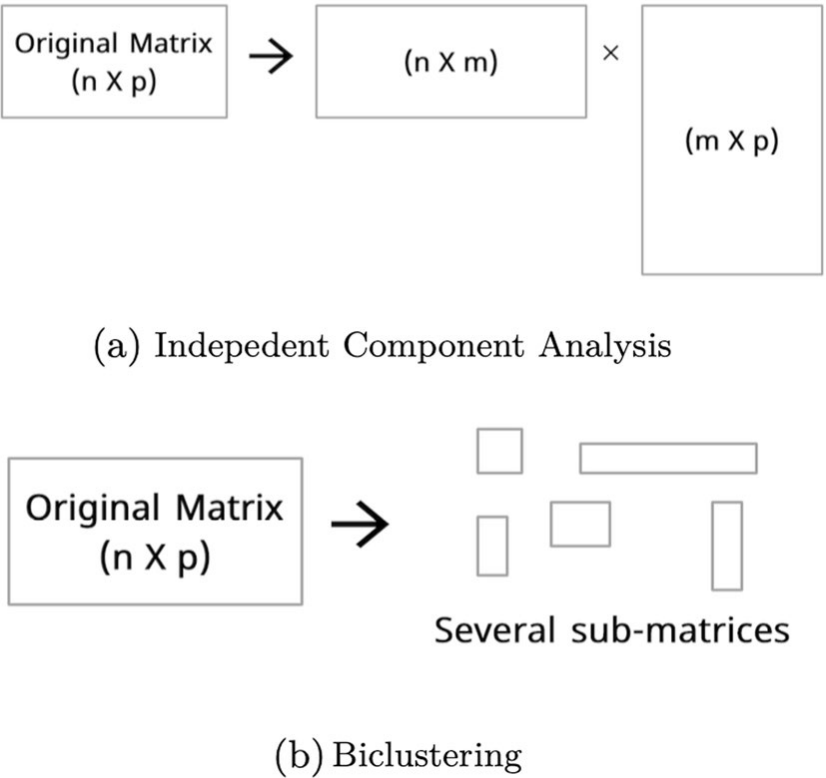
Differences between ICA and Biclustering. While ICA decomposes the original matrix, biclustering generates an arbitrary number of sub-matrices (depending on the algorithm)

ICA and biclustering are fundamentally different techniques. While ICA transforms a $n\times p$ matrix into matrices $n \times m$ and $m \times p$, where *m* is the number of components (arbitrary chosen). Biclustering operates on a $n \times p$ matrix and extracts an arbitrary number of sub-matrices with an arbitrary size (Fig. 14 illustrates the differences between biclustering and ICA).

While ICA and biclustering are fundamentally different algorithms, decomposition techniques are used as an intermediate step in the internall bicluster algorithm [35, 37, 38, 78, 79]. An additional approach follows Gupta et al. [29], who proposed a new biclustering approach in the **Subject** × **Voxel** dimensions to analyse structural MRI data, using an intermediate ICA step.

An interesting application of ICA is its capacity to connect to other analysis techniques such as clustering in an analysis pipeline. ICA is used as a preprocessing step, and a clustering approach operates in the results of ICA [80–82]. The same approach can be done with biclustering: instead of applying biclustering directly on fMRI time series (the approach of this study), biclustering algorithm can operate on the ICA results [14].

#### Graph theory

Graph theory is a mathematical field, applied in neurosciences to characterize the network structure, and models the brain as a set of vertices (which represent either ROIs or even single voxels) and the connections between them as edges. Graph techniques can be used either to analyse individual vertices or the graph as a whole [3]. Heuvel et al. [83] and Rubinov et al. [84] discuss the use of graph theory to analyze the brain network, in particular the use of fMRI time series to construct the brain network. This approach to analyse fMRI data has been used in the past, not only to cluster the brain [85, 86], but also in tasks such as detecting schizophrenia [87].

**Fig. 15:**
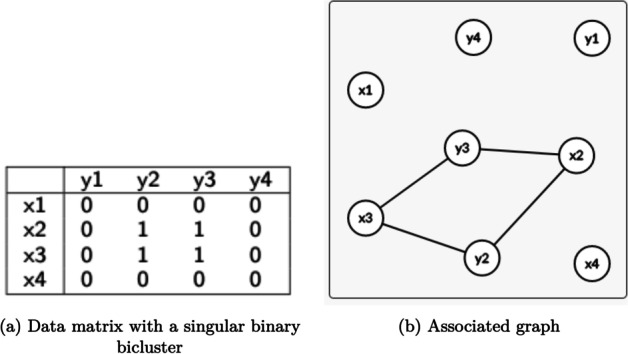
Relation between biclustering and graph theory: a biclustering can be seen as a submodule in a network

Madeira and Oliveira [7], and Henriques et al. [33] established a theoretical connection between biclustering and graph theory. A data matrix can be seen as a weighted bipartite graph, and biclustering of a data matrix is conceptually equivalent to discovering of maximal cliques or other structures from graphs obtained from binary or real-valued matrices. This connection motivates the use of biclustering to find maximal cliques or other structures from graphs obtained from binary or real-valued matrices, where edge values identify connection strength [88, 89]. Several biclustering algorithms use graph concepts internally to obtain biclusters [90–94]. Figure 15 illustrates the conection between biclustering and graph theory.

Biclustering has been extensively used in gene expression data analysis to uncover graph structures such as the discovery of co-expressed gene modules and regulatory networks. A review on the application scenarios of biclustering in the discovery of biological networks is provided by Xie et al. [9].

#### Classification tasks

A group of studies has been exploring fMRI techniques to study neurologic and psychiatric brain disorders, suggesting that neurodegenerative diseases target cortical networks rather than single regions [95]. These approaches have been applied in several diseases such as Alzheimer’s [96, 97], depression [98], dementia [99], multiple sclerosis [100], amyotrophic lateral sclerosis [101] and schizophrenia [87, 102–105]. In general, the goal of biomedical research is to establish clinical biomarkers. These biomarkers are a set of characteristics (features) that allows early disease detection and prognostic prediction [106–110].

**Fig. 16:**
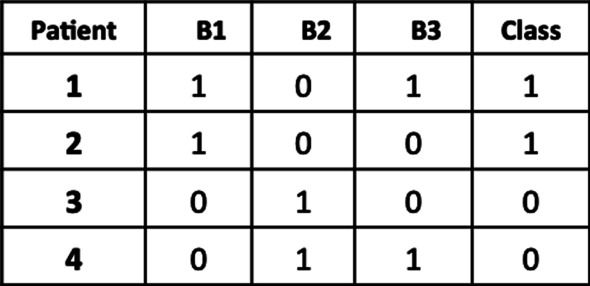
After discovering the biclusters for a group of subjects, a data matrix can be obtained locating the presence of some bicluster in a subject, and then used for classification tasks. The biclusters (sets of features and corresponding representative values) are used as features (Figure adapted from [112])

Biclustering has been used for supervised learning tasks [111–113]. Since a bicluster is representative of a certain characteristic of a subject, the presence of a bicluster can be used as a biomarker to discriminate characteristics of the population. Figure 16 illustrates this approach.

Bicluster based classification have been used previously for both temporal and non-temporal data. Considering temporal data, Carreiro et al. [111] considers biclusters of $Genes\times Time$ to identify biological processes related to the progression of Multiple Sclerosis patients. This approach was extended, considering not biclusters, but meta-biclusters (clusters of biclusters) [113]. In these approaches, the biclustering algorithm *CCC* was used. Matos et al. [112] used the *BicPAM* biclustering algorithm to analyze non-temporal data, together with the concept of meta-biclusters to characterize amyotrophic lateral sclerosis patients. Recently, Henriques and Madeira [114] showed that the use of biclustering based classification improves the performance of state-of-the-art classifiers.

## Conclusions

Our study evaluated the use of biclustering in the context of fMRI data. Seven state-of-art biclustering algorithms were selected, compared among each other and with three traditional clustering algorithms. Our results show that most biclustering methods are not able to clearly surpass the traditional clustering when using the virtual error metric. However, the exhaustive methods (BicPAM and CCC) are able to achieve the best values of coherency of all tested algorithms. Additionally, and independently of the measured homogeneity, we point out that biclustering can be useful, and an improvement in comparison to clustering, due to its ability to consider both spatial and temporal dimensions to discover the groups. We also observed that the bicluster structures found in this type of data are of a shifting nature.

Focusing on individual performance, BicPAM achieved high homogeneity levels by generating many small biclusters, which is not consistent with the promise of an exhaustive search. The issue is related to the performance of the algorithm, since it needs a prohibitive quantity of time and resources to discover bigger biclusters. This could be solved considering two approaches: the first one is related to the algorithm that can be optimized to generate results faster; the second is related to the parameterization of the algorithm that could be used if the size of the desirable biclusters is known apriori. It must be noticed that these observations are not inconsistent with the obtained results: even if this is considered, BicPAM will still be able to achieve good levels of homogeneity in its best biclusters.

Another interesting result comes from the greedy biclustering solutions, ISA and XMotifs. While not being able to achieve results as impressive as the exhaustive ones, they are still able to find some good ones using a fraction of the time BicPAM needs to operate. Additionally, while ISA works by using directly the real data, XMotifs requires a discretization step, which allows different approaches.

Considering the trade-off between the number of generated biclusters, their quality and execution time, Bimax is an interesting choice since it finds a huge number of biclusters very fast. This is achieved by doing a binarization of the data. While not being able to detect the best biclusters, its velocity could simply mean that the algorithm could be used to get some insights on the expected biclustering structures before running other algorithms.

FABIA and Spectral Biclustering are not able to achieve interesting results. Despite being both based on the same search type (Distribution Parameter identification) we believe that the reasons for this are different. The search strategy of FABIA means that the number of solutions will be limited to the number of columns of the dataset, which will strongly limit its capacity to generate solutions with many biclusters. Spectral Biclustering on the other hand is limited due to the generated checkerboard bicluster structure.

The last algorithm considered is CCC which uses notions of temporal contiguity during the exhaustive search for biclusters. This means that CCC is able to find multidimensional time series motifs. This leads to the generation of a high number of biclusters with an easy interpretation which could be possibly ideal for these types of analyses. Since time is contiguous in these biclusters, a new set of quality measures based on statistical significance can further be used for an improvement when filtering results. Furthermore, the high coherency levels observed in this study motivate the use of specific temporal biclustering methods to study fMRI data.

Biclustering is a tool to search for local patterns in data well established and recognised in the gene expression application context. Our study shows that biclustering is equally promising in fMRI data. While comparative studies provide guidance over the selection of the methods, the choice of the biclustering algorithm to analyse must be guided by the study objectives.

## Data Availability

The data used for the paper, as well as auxiliary scripts are available at https://github.com/ECastanho/Biclustering-fMRI-time-series-a-comparative-study.

## Abbreviations

fMRI: Functional magnetic resonance imaging
EEG: Electroencephalography
MEG: Magnetoencephalography
GO: Gene ontology
KEGG: Kyoto Encyclopedia of Genes and Genomes
MSR: Mean squared residue
SMSR: Scaling mean squared residue
VAR: Variance
VE: Virtual error

## Footnotes

1 Original data available at: acgt.cs.tau.ac.il/twigs/.

2 Original data available at the openneuro portal: openfmri.org/dataset/ds000102/.
